# Conserved Epigenetic Mechanisms Could Play a Key Role in Regulation of Photosynthesis and Development-Related Genes during Needle Development of *Pinus radiata*

**DOI:** 10.1371/journal.pone.0126405

**Published:** 2015-05-12

**Authors:** Luis Valledor, Jesús Pascual, Mónica Meijón, Mónica Escandón, María Jesús Cañal

**Affiliations:** 1 Plant Physiology, Faculty of Biology, University of Oviedo, Cat. Rodrígo Uría s/n, E-33071, Oviedo, Spain; 2 Department of Biology and CESAM, University of Aveiro, Campus Universitario de Santiago, P-3810-193, Aveiro, Portugal; 3 Regional Institute for Research and Agro-Food Development (SERIDA), Finca Experimental La Mata s/n, E-33825, Grado, Spain; Universidad Miguel Hernández de Elche, SPAIN

## Abstract

Needle maturation is a complex process that involves cell growth, differentiation and tissue remodelling towards the acquisition of full physiological competence. Leaf induction mechanisms are well known; however, those underlying the acquisition of physiological competence are still poorly understood, especially in conifers. We studied the specific epigenetic regulation of genes defining organ function (Pr*RBCS* and Pr*RBCA*) and competence and stress response (Pr*CSDP2* and Pr*SHMT4*) during three stages of needle development and one de-differentiated control. Gene-specific changes in DNA methylation and histone were analysed by bisulfite sequencing and chromatin immunoprecipitation (ChIP). The expression of Pr*RBCA* and Pr*RBCS* increased during needle maturation and was associated with the progressive loss of H3K9me3, H3K27me3 and the increase in AcH4. The maturation-related silencing of Pr*SHMT4* was correlated with increased H3K9me3 levels, and the repression of Pr*CSDP2*, to the interplay between AcH4, H3K27me3, H3K9me3 and specific DNA methylation. The employ of HAT and HDAC inhibitors led to a further determination of the role of histone acetylation in the regulation of our target genes. The integration of these results with high-throughput analyses in *Arabidopsis thaliana* and *Populus trichocarpa* suggests that the specific epigenetic mechanisms that regulate photosynthetic genes are conserved between the analysed species.

## Introduction

Leaf development is a complex process that involves many cell and tissue differentiation and remodelling processes [1]. During this period, two different stages can be observed: the proliferative stage, which covers the initial period of development and is characterised by low cell differentiation and a high rate of cell division that will define the organ layers and shape, and the expansion stage characterised by cell expansion and differentiation and ending with the gain of physiological competence and maturity [2–5]. Despite the gene interaction networks and regulatory cascades which lead to leaf primordium initiation and organ shape formation are mostly defined [6–10], the mechanisms for activating the biochemical pathways that define organ functions are still poorly understood.

In *Pinus radiata*, needles reach maturity after 12 months of development [11]. During this period primary metabolism is directed towards photosynthesis and other biosynthetic pathways, that will support normal tree growth and function while, at the same time, growth-related activities such as increased protein and structural biosynthesis, cell division and morphogenesis become down-regulated or completely silenced [12].

It is clear that organ induction, development, and maturation are the consequence of highly specific regulatory mechanisms that control gene expression and protein modification and degradation, required for successful organ development [4,6,10,13]. Among these mechanisms, epigenetic regulation is one of the most determinant regulatory pathways [14–16]. DNA methylation and histone post-translational modifications (PTMs) have been revealed as key mechanisms for controlling chromatin structure and function [17] and regulating cell growth and differentiation [18–22]. These mechanisms are dynamic and can consequently be reverted or adapted to particular environmental situations, constituting a link between genotype and phenotype [23].

Despite the availability of genome-wide mapping of DNA methylation and histone PTMs in *Arabidopsis thaliana* (Arabidopsis), *Oryza sativa* (Rice), and *Populus trichocarpa* (Poplar) [22,24–26] and the common consensus of the permissive (AcH4, H3K4me3) and repressive (H3K9me3 and H3K27me3) effects of histone PTMs at the transcriptional level [17,27], in-depth studies of the gene-specific epigenetic mechanisms involved in the control of plant organ differentiation and maturation are still limited. Focusing on leaves, previous studies have shown that some leaf morphogenesis-related genes such as *FLOWERING LOCUS C* (*FLC)*, KNOTTED1-LIKE HOMEOBOX (KNOX) family and *SHOOT MERISTEMLESS (STM)* [6,28,29] or the carbon-concentrating mechanism-related genes *phosphoenolpyruvate carboxylase* (*PEPC)* and *MALIC ENZYME (ME)* [30] are epigenetically regulated. The transition from proliferative growth to the expansion and differentiation stages increases global DNA methylation and causes changes in histone PTMs in Arabidopsis [31]; however, the specific regulation of enzymes related to primary and secondary metabolism at any developmental stage or environmental situation is still poorly studied and has only been addressed in a small number of high-throughput analyses. Charron et al. [32] analysed the landscape of H3K9ac, H3K9me3, H3K27ac, and H3K27me3 during the de-etiolation process in Arabidopsis, showing a novel insight into the epigenetic regulation of a specific physiological process. Recently, Lafos et al. [33] provided an overview of H3K27me3 during Arabidopsis de-differentiation, pointing to the importance of this repressive mark via a comparison of stem and leaf differentiated cells.

A deeper knowledge of the epigenetic regulation of key pathways involved in maturation and plant survival, such as carbon fixation and stress responses, and its potential reversion, has a direct biotechnological application in clonal breeding, since ageing and maturation is the most important barrier in clonal forestry programmes [34]. To date, efforts towards the characterisation of the epigenetic role of tree cell plasticity and in growth and development processes have been focused on global epigenetic dynamics for defining phase change, maturation stage, bud set and burst, and production hallmarks (see Bräutigam et al. [21] for a review), with no record to our knowledge, of any study dealing with gene-specific epigenetic dynamics during leaf maturation.

To fill this gap we have studied four genes that had demonstrated a differential accumulation of its products during needle development [12] and have a major function in needle development and plant growth covering photosynthesis (*RUBISCO ACTIVASE*, PrRBCA; *RUBISCO SMALL SUBUNIT*, Pr*RBCS*), one carbon metabolism (*SERINE HYDROXYMETHYL TRANSFERASE 4*, Pr*SHMT4*) [35], and developmental control and stress response (*COLD SHOCK DOMAIN PROTEIN 2*, Pr*CSDP2*) [36]. We cloned these genes and investigated their transcription levels in three needle developmental stages and a de-differentiated tissue (white, non photosynthetic, calli derived from needles which is showing active growth) used as control. Moreover, we described the epigenetic regulation mechanisms acting on the promoter and first exon sequences either by cytosine methylation, and/or by specific histone PTMs AcH4, H3K4me3, H3K9me3 and H3K27me3. The employ of inhibitors of histone deacetylases (HDACs) and histone acetyltransferases (HAT) provided a deeper understanding of the role of AcH4. These results provide new insights over how the epigenetic mechanisms regulate key metabolic pathways during needle maturation.

## Materials and Methods

### Plant material and treatments

**Fig 1:**
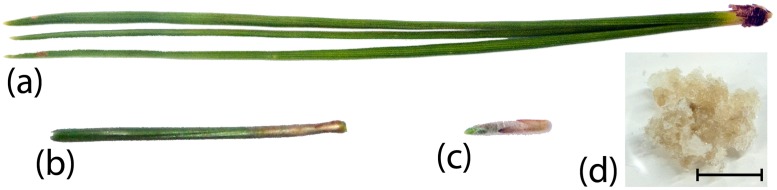
Different developmental stages analyzed in this work: B12 mature needles (a), B5 developed needles (b), B1 immature needles (c) and calli (d) were used as a control of de-differentiated tissue with high growth capacity. Horizontal bar represents 1 cm length.

Needles that were either fully developed and mature (12-month-old; B12), those that were developed but in transition between cell proliferation and expansion (5-month-old; B5), or immature (three- to five-week-old; B1) (Fig 1a–1c), were collected from three different adult *Pinus radiata* trees grown in a test-garden of the University of Oviedo during the active growth season (spring), washed with tap water, dried with filter paper, excised from the tree and frozen *in situ* in liquid nitrogen. Samples were stored at -80°C prior to DNA or RNA extraction.

*Pinus radiata* callus (Fig 1d) was employed as a de-differentiated control. Calli were induced from explants taken from the same trees from which needles were sampled. After surface sterilisation of the explants, callus was induced in EDM6 medium [37] supplemented with 9 μM 2,4-Diclorophenoxyacetic acid and 2.66 μM 6-Benzylaminopurine and grown in the dark at 25°C. Once initiated, calli were subcultured every 15 days in the same medium and conditions.

One-year-old seedlings of *Pinus radiata* grown in greenhouse were treated in independent blocks of 3 individuals with Suberoylanilide Hydroxamic Acid (SAHA) (SML0061-5MG, Sigma), an inhibitor of HDACs [38]; Anacardic Acid (AnAc) (A7236-5MG, Sigma), an inhibitor of HATs [39]; and a mock control (9 plants were considered for this assay). Drugs were dissolved in DMSO and then diluted in _dd_H_2_O to 200 μM. We performed previous dosage trials and this concentration was previously proved to be efficient for slight modifications of the epigenome without altering cell viability in cell cultures (data not shown). Drugs were applied in solution to the media for the calli and by nebulization over the needles for the seedlings. Seedlings were treated twice, with a gap of 48 h between re-treatment. Plant material was sampled after 48 h of the last treatment, flash-frozen, and kept at -80°C until use.

### Combined isolation of DNA, RNA, and Proteins

The different biomolecules were extracted from the same samples employing a sequential fractionation as described in Valledor et al. [40]. In brief, samples were ground to powder in liquid nitrogen and washed with chloroform:methanol:water (1:2.5:0.5). After centrifugation, supernatants were discarded, and resulting pellets were resuspended in 7M Guanidine HCl, 2% (v/v) TWEEN 20, 4% (v/v) NP-40, 50 mM Tris, pH 7.5, 1% (v/v) ß-mercaptoethanol. DNA was purified from the mixture by filtering the solution through a silica mini-spin column (EconoSpin, Epoch Life Science). 300 μL of acetonitrile were then added to the flow-through containing RNA and proteins, and RNA was isolated from the solution by filtering through a new mini spin column. Spin columns were washed with 600 μL of 2 mM Tris pH 7.5, 20 mM NaCl, 0.1 mM EDTA, 90% ethanol, and then 600 μL of 2 mM Tris pH 7.5, 20 mM NaCl, 0.1 mM EDTA, 70% ethanol. Nucleic acids were eluted from the columns by adding 50 μL of _dd_H_2_O. Nucleic acids were quantified by spectrophotometry and its integrity was checked in denaturing agarose gels. RNA samples were treated with DNase I (Fermentas) and its possible contamination with DNA was checked by PCR.

Proteins were recovered by mixing the flow-through with 2 volumes of phenol:water (1:1). Samples were then centrifuged at 10000 x g, and organic phase was recovered. Proteins were precipitated by adding 2 volumes of 0.1 M ammonium acetate in methanol. After an overnight incubation at -20°C, tubes were centrifuged, and protein pellets were washed twice with acetone. Dry pellets were dissolved in 4% SDS, 8 M Urea and quantified using BCA assay.

### RT-qPCR expression analyses

One μg of total DNA-free RNA and Superscript III first-strand cDNA synthesis kit (Invitrogen) were used for first strand cDNA synthesis previous to quantitative PCR. Quantitative PCR was performed as followed: 10 ng cDNA, 5pM of each primer and Perfecta SYBR Green (Quanta) were mixed and amplified in ABI 7900HT system. Three measurements for each transcript and sample were analyzed using Prism software (Applied Biosystems). Relative quantifications were performed for all targets employing the expression levels of pine *ACTIN* (AY172979.1), *18S* (M82462.1) and *GAPDH* (KM496531.1) genes as loading controls. Primer sequences are listed in S1 Table and were designed according to described GenBank sequences.

### Amplification of 5’ cDNA ends of candidate genes

The available sequences for the candidate genes *PrRBCA* (dbEST GO096305), *PrRBCS* (dbEST TC92228), *PrSHM4* (dbEST GO270978), and *PrCSDP2* (dbEST AA216460) were amplified towards 5’ employing RML-RACE kit (Ambion) following the instructions provided by the manufacturer. Amplified sequences were extended towards promoter using a chromosome walking kit (Genome Walker Universal Kit, Clontech). The obtained sequences were included as supplemental material (S1 File).

### Analysis of global DNA methylation

Global DNA methylation was quantified by high performance capillary electrophoresis (HPCE) as previously described [41]. Quantification of the relative methylation of each DNA sample was calculated as follows:

$$\%\text{5}mdC\;=\frac{\text{5}mdC\;Peak\;Area}{\text{5}mdC+dC\;Peak\;Areas}\times \;100$$

### Analysis of sequence-specific DNA methylation

The methylation status of specific genomic DNA sequences was established by bisulfite genomic sequencing. A total of 1.8 μg of genomic DNA were bisulfite converted by using EpiTect Bisulfite kit (Qiagen). Three different biological replicates per developmental stage were processed to minimize potential artefacts and for ensuring conversion reproducibility. Once DNA was converted, target sequences were amplified employing specific primers. PCR product was cloned into pGEM-T easy (Promega). Eight colonies were sequenced for each gene and sample to measure the methylation status of every cytosine. Cytosine-rich regions were detected employing jEMBOSS 1.5 CpG plot utility (EMBOSS), and CNG and CNN sites were determined manually. Cytosine-rich regions were only detected in the *PrCSDP2* gene (S1 Fig). Primer sequences and PCR conditions for methylation analysis are indicated in S1 Table.

### Western blot

Twenty-five μg of protein were separated by electrophoresis in 15% acrylamide SDS gels and then transferred by electroblotting (350mA for two hours) to Immobilon membranes (Millipore). Membranes were blocked with skimmed milk and histone marks were detected with anti-acetyl-Histone H4 (Upstate, ref. 06–866), anti-trimethyl-Histone H3 (Lys4) (Upstate, ref. 07–473), anti-trimethyl-Histone H3 (Lys9) (Upstate, ref. 07–442) or anti-trimethyl-Histone H3 (Lys 27) (Upstate, USA, ref. 07–449) and polyclonal anti-delta-tubulin (Chemicon, ref. AB3203); the latter was used as a control for protein loading [42]. Secondary antibody was coupled to alkaline phosphatase (Calbiochem, ref. 401312). Membranes were digitized and analysed employing Fiji [43], characteristic membranes are provided in S2 Fig. Relative abundance (RA) of each histone mark was calculated as follows:

$$RA\;=\frac{Histone\;mark\;band\;intensity}{Tubulin\;band\;intensity}\times \;100$$

### Chromatin immunoprecipitation (ChIP)

Samples were crosslinked by incubating 2 g of fresh tissue during 16 min *in vacuo* with cross-linking solution (0.4 M sucrose, 10 mM Tris-HCl pH 8.0, 1 mM EDTA pH 8.0, 2% formaldehyde). Reactions were quenched with 2 mL 2.5M glycine after 14 min. Nuclei were isolated according to Haring et al. [44] and chromatin was sonicated until fragments of an average length of 0.3–0.6 kb were obtained. Five μg of sonicated chromatin were diluted to a final volume of 1 ml in ChIP dilution buffer (1.1% Triton X-100, 1.2 mM EDTA, 167 mM NaCl, 16.7 mM Tris-HCl pH 8, 1mM PMSF). Chromatin was precleared by adding 25 μL of Dynabeads (Invitrogen). After 2 hours of incubation at 4°C Dynabeads were removed. For each test, 5 μL of antibody (see above for references) or _dd_H_2_O (NoAb), were added to pre-cleared solutions and incubated for 10 h at 4C. Inmunocomplexes were purified with 25 uL of Dynabeads and washed first in low salt buffer (150 mM NaCl, 20 mM Tris-HCl pH 8.0, 1% SDS, 1% triton X-100, 2 mM EDTA), then in high salt buffer (500 mM NaCl, 20 mM Tris-HCl pH 8.0, 0.1% SDS, 0.5% triton X-100, 2 mM EDTA), LiCl buffer (250 mM LiCl, 10 mM Tris-HCl pH 8.0, 1% triton X-100, 1% sodium deoxycholate, 1 mM EDTA) and finally in 1 mL of TE buffer (10 mM Tris-HCl pH 8.0, 1 mM EDTA). Immunocomplexes were eluted by adding 250 μL of ChIP elution buffer (1.0% SDS, 0.1 M NaHCO_3_), and crosslinking was reversed by adding 10 μL 5 M NaCl and incubating for 4 hours at 65°C. Input fraction was constituted by adding 5 μg of sonicated chromatin to 1 mL of ChIP elution buffer. DNAs ware purified and PCR amplification was performed as described in S1 Table.

### Statistical analyses

Data (global DNA methylation and histone marks, gene expression data) were subjected to analysis of variance (ANOVA) using the R version 2.9.2 software [45]. Normality and homoscedasticity were evaluated by Shapiro—Wilk and Levene tests respectively. Multiple comparisons were performed by significant Tukey’s HSD test. A probability level of p< 0.05 was considered significant for all statistical analyses. Five biological replicates were analysed. Quantitative PCR data was processed according to Hellemans et al. [46] and R packages EasyqPCR and SLqPCR before applying ANOVA.

## Results

### Development induces changes in the expression level of candidate genes

**Fig 2:**
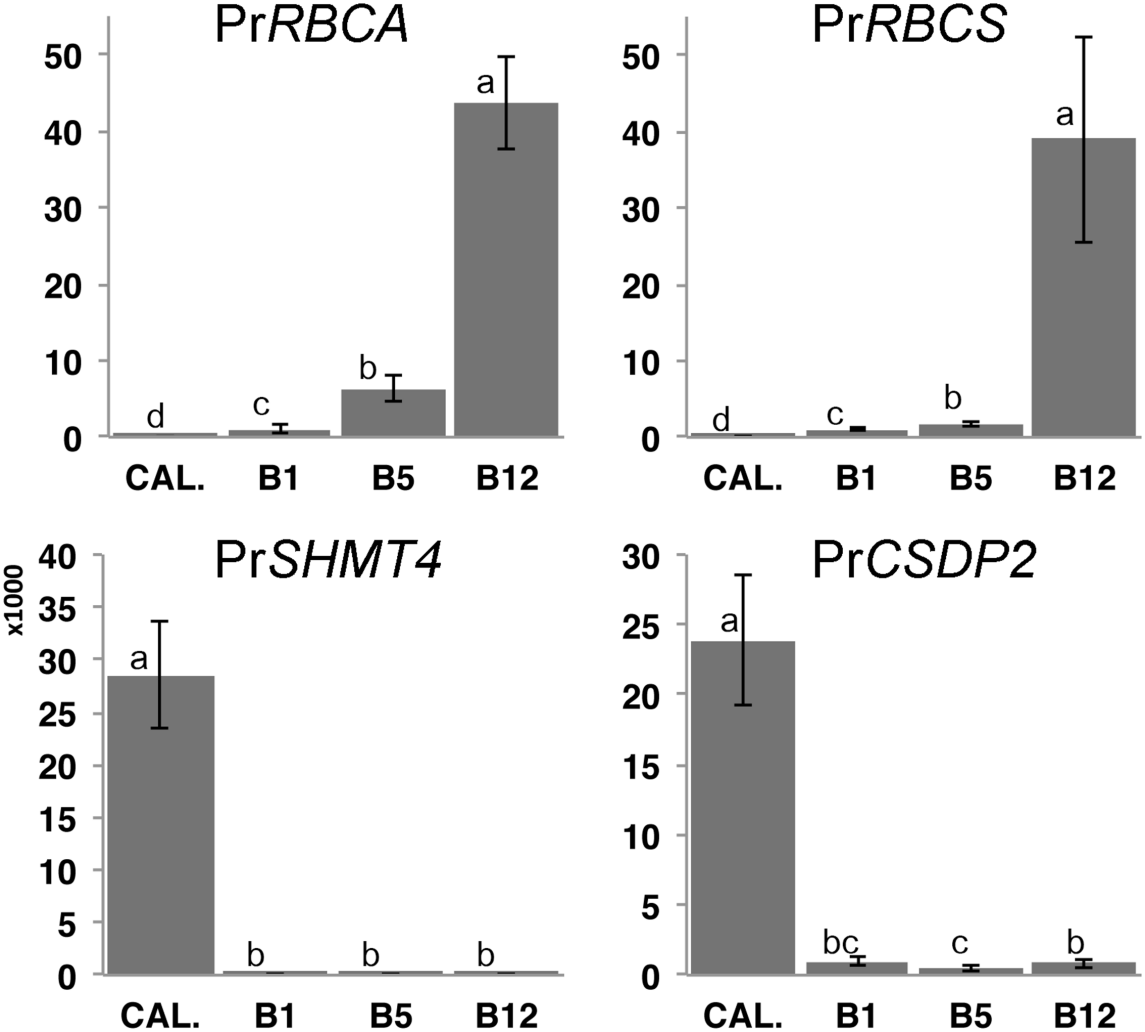
Analysis of the Relative Quantity (RQ) of Pr*RBCA*, Pr*RBCS*, Pr*SHMT4*, and Pr*CSDP2* expression levels in different tissues obtained by RT-qPCR. Values were normalized and expressed as fold differences compared to B1 needles. Error bars show the propagated standard error of RQ, SE(RQ). Different letters between bars corresponding to each gene indicate significant expression differences between developmental stages (ANOVA followed by a Tukey HSD test; p < 0.05).

To assess how epigenetic mechanisms might regulate needle development and maturation in *Pinus radiata*, we sampled needles at different developmental stages (B1 first stages of division from needle primordia, B5 transition between proliferative and expansive stage, B12 mature needles) and generated de-differentiated callus tissue to serve as a non-photosynthetic pluripotent control (See methods; Fig 1). We selected two genes closely related to leaf maturation; Pr*RBCA*, Pr*RBCS*, and two genes related to immature tissues Pr*SHMT4* and Pr*CSDP2*, for which partial sequences were available. These genes showed different expression levels in the different tissues analyzed (Fig 2): Pr*RBCA* and Pr*RBCS* increased expression levels with leaf maturation, being this transcript not detected in callus and showing a low expression level in B1 needles. As expected, in a developing tissue acquiring photosynthetic ability (B5), the Pr*RBCA* expression level was higher than that of Pr*RBCS*. Alternatively, Pr*CSDP2* was highly expressed in callus tissue. B1 showed the highest expression level of Pr*CSDP2* between the different needles and Pr*SHMT4* was only expressed in calli.

### Needle developmental processes are associated with changes in global epigenetic markers

**Fig 3:**
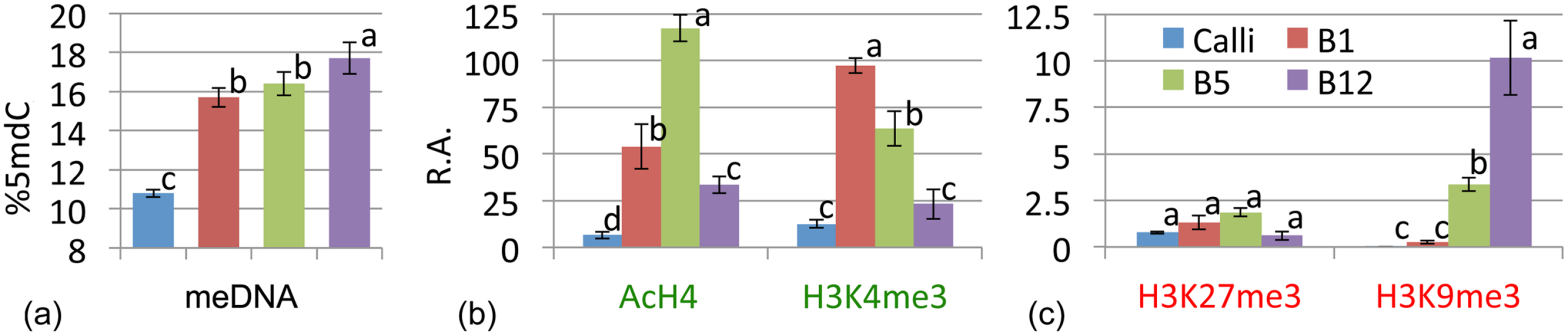
Analysis of global DNA methylation in the different needle developmental stages. (a). Relative abundance (R.A.) of the permissive, AcH4 and H3K4me3 (b), and repressive, H3K27me3 and H3K9me3 (c) histone marks in the indicated developmental stages. Different letters between bars corresponding to each histone PTM indicate significant expression differences between developmental stages (ANOVA followed by a Tukey HSD test; p < 0.05).

Tissue differentiation was associated with an increased level of global DNA methylation (Fig 3a). Calli showed a low degree of DNA methylation, of 10.8%, while in B1 and B5 needles it progressively increased to 15.7% and 16.4% respectively. B1 needles were rapidly developing and were characterised by a large increase in cell number, whereas B5 needles had fewer cell divisions and showed an increase in differentiation. The mature B12 needles showed the highest degree of DNA methylation at 17.7%.

The analysed histone PTMs showed different abundances, and were tissue- and modification-specific. AcH4 showed a 2.5-fold increase in B5 needles compared to B1 needles and the lowest levels were in the differentiated tissues of B12 needles; surprisingly, calli had the lowest abundance of this mark, whereas the other permissive mark, H3K4me3, showed different dynamics. This result demonstrates the complexity of epigenetic regulation mechanisms. The abundance of H3K4me3 was highest in B1 needles and was negatively correlated with needle differentiation; calli showed the lowest levels of this mark (Fig 3b). H3K27me3 showed no significant differences between developmental stages, but H3K9me3 abundance progressively increased, with a maximum in B12 needles.

After 5’ *de novo* sequencing of target genes, only cytosine rich region in the first exon of *CSDP2* gene was identified (S1 Fig). In consequence only *CSDP2* was further analysed for changes in specific DNA methylation whereas all genes were subjected to ChIP-PCR.

### Pr*CSDP2* was regulated epigenetically by specific DNA methylation and specific patterns of histone modifications in the first exon region but not in the promoter

**Fig 4:**
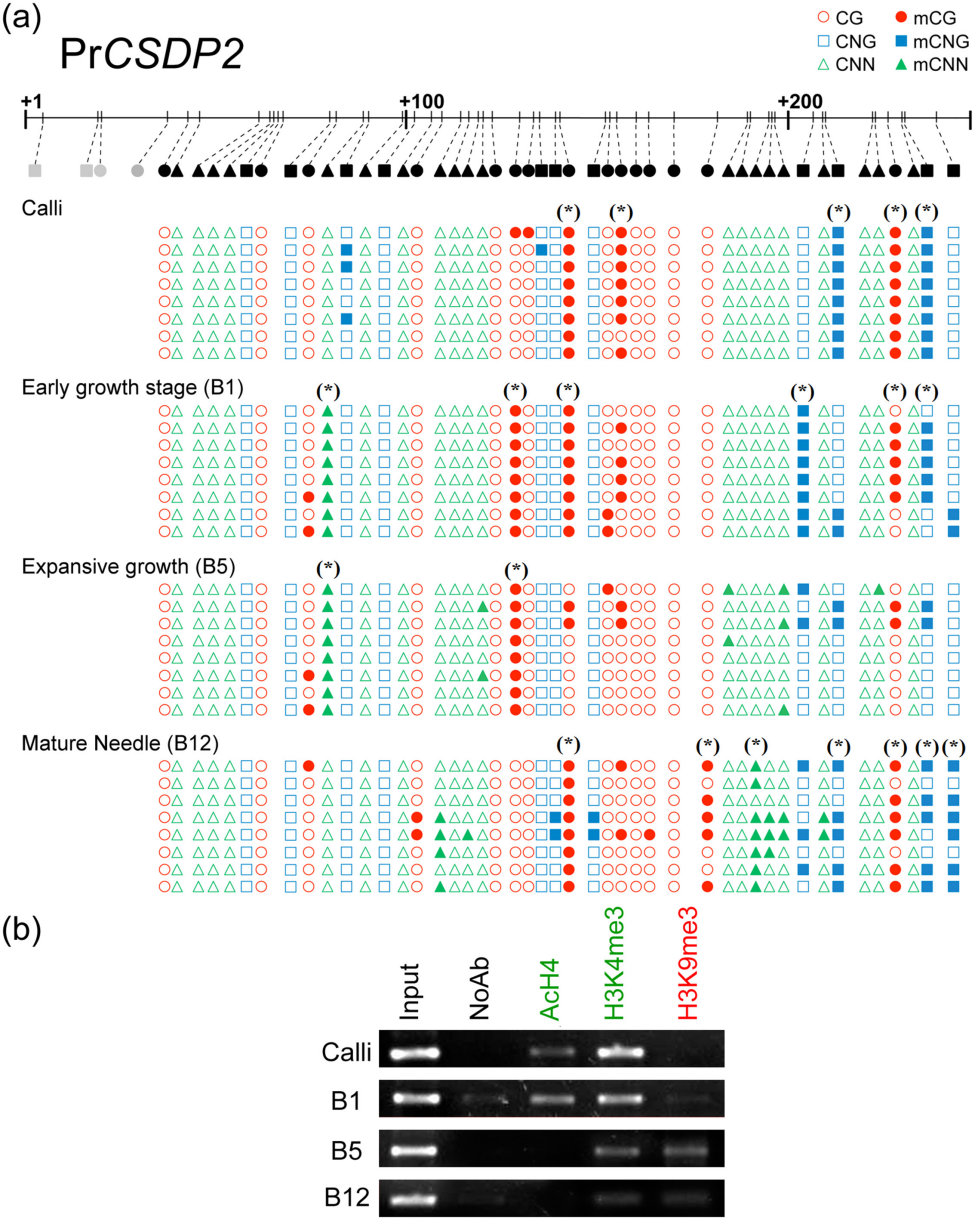
(a) Bisulfite methylation analysis of Pr*CSDP2* in callus, growing needle and mature needles. Approximately 250 bp of the first exon were sequenced. Cytosines in grey were present in the indicated region, but not analyzed (b) Effects of the needle developmental stage over the ChIP-based enrichment of the first exon of Pr*CSDP2* using the indicated anti histone-PTMs antibodies. Image corresponds to a representative gel electrophoresis using primers for amplifying a region corresponding to the promoter of Pr*CSDP2* gene.

We observed differential patterns of DNA methylation after bisulfite sequencing the previously defined cytosine rich region of Pr*CSDP2* (Fig 4a). Calli showed the greatest number of methylated cytosines, with five heavily methylated residues. B1 needles showed a lower overall number of methylated cytosines but the number of methylation loci increased to 11, indicating *de novo* methylation events and targeted demethylation compared to calli. B5 showed a transitory landscape, whereas B12 needles showed the highest number of methylated loci, with seven from 19 being heavily methylated.

ChIP studies revealed an enrichment of AcH4 in the first exon of the Pr*CSPD2* gene (Fig 4b) in calli and B1 that was not detected in B5 and B12 needles. The enrichment of the H3K4me3 fraction was also observed in less differentiated tissues and the enrichment of H3K9me3 was positively correlated with needle development. These results correlate with the expression levels described for this gene, which decrease with an increase in tissue differentiation, and are supported by the fact that the application of HDAC or HAT inhibitors altered the expression pattern of this gene (see below). The promoter region did not show significant epigenetic changes between the different developmental stages (data not shown).

### The level of H3K9me3 in the promoter is correlated to the expression of Pr*SHMT4*

**Fig 5:**
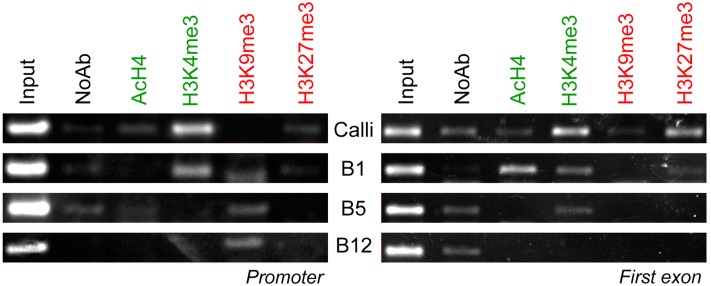
Effects of the needle developmental changes upon the ChIP-based enrichment of chromatin fractions corresponding to the promoter or first exon of Pr*SHMT4* using the indicated anti histone PTMs antibodies.

Pr*SHMT4* transcription was only detected in calli and was repressed in needles (Fig 2). As is described for the other genes, the promoter and the first exon regions behaved differentially. Focusing on the promoter, calli are characterised by a strong enrichment of the H3K4me3 marker and also by the presence of AcH4 above the control threshold (Fig 5). An absence of the AcH4 mark, and the enrichment of the H3K9me3 fraction, correlates with the silencing of Pr*SHMT4* in B1 needles. B5 and B12 needles only exhibited repressive marks in the promoter. The first exon showed a similar behaviour to that of the promoter, with a predominance of H3K4me3. However, we were unable to detect strong repressive marks in this region at all developmental stages.

### Pr*RBCA* and Pr*RBCS* are regulated by histone post-translational modifications at the promoter level

**Fig 6:**
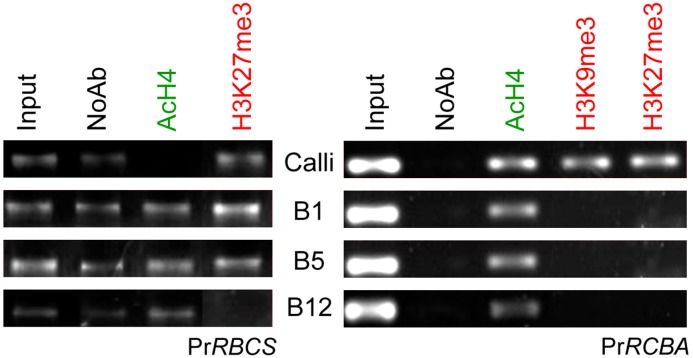
Effects of the needle developmental changes upon the ChIP-based enrichment corresponding to chromatin fractions of the promoters of Pr*RBCS* and Pr*RBCA* genes using the indicated anti histone PTMs antibodies.

The interplay between AcH4 and H3K27me3 appears to play an important role in the regulation of expression of the studied photosynthetic genes. In calli, a non-green heterotrophic tissue in which Pr*RBCA* and Pr*RBCS* are repressed, strong epigenetic silencing marks were found in the promoters (Fig 6). In the case of Pr*RBCS*, the presence of AcH4 in green autotrophic tissues and also one repressive mark like H3K27me3, which is lost in mature needles, appeared to explain the progressive increase in the expression levels of this gene throughout development, and its silencing in calli (H3K4me3 and H3K9me3 fractions did not show significant differences between developmental stages). The Pr*RBCA* promoter showed an enrichment of the AcH4 histone fraction in all studied material; however, an enrichment of the repressive marks H3K9me3 and H3K27me3 was only found in calli, demonstrating the importance of these marks for defining green photosynthetic and non-photosynthetic tissues. However, we did not observe any difference between histone marks within the first exon at different needle developmental stages, which suggests that the epigenetic regulation of these genes occurs via promoter region.

### The exogenous application of exogenous HATs and HDACs inhibitors altered gene expression levels of target genes in calli and seedlings

The application of AnAc (inhibits the binding of the HAT activators p300 and p300/CBP-associated factor, resulting in a decrease of acetylated histones [47]) or SAHA (chelates Zn^-^ ions at active centre of HDACs, resulting in the accumulation of acetylated histones [48]) at low concentration resulted in an altered gene expression in calli (S3A Fig). Both AnAc and SAHA increased the expression of Pr*CSDP2*. On contrary, AnAc reduced Pr*RBCA* expression, while SAHA did not have any effect. Acetylation drugs didn’t alter the expression of Pr*RBCS* and Pr*SHTM* so we hypothesize a possible regulation by specific histone methylation marks. In needles (S3B Fig) the treatment with SAHA increased the abundance of Pr*RBCS* but reduced the expression levels of Pr*RBCA* and Pr*SHMT4* probably because SAHA has pleiotropic effects over other regulatory network linked to these genes. On the other hand the low permeability of needles to AnAc probably reduced the effects of this compound as it is shown in S3B Fig.

## Discussion

### The expression levels of Pr*RBCA*, Pr*RBCS*, Pr*CSDP2*, and Pr*SHMT4* can be used to distinguish between needle maturation stages

Leaf development is a complex process in which different systems act co-ordinately to acquire full physiological competence. In Arabidopsis, these processes are divided into two different stages: proliferative, characterised by a large number of cell divisions, and expansion, in which cells expand and reach physiological maturity [3,5]. Each stage is further characterised by the activation or repression of different molecular mechanisms involved in the different process. The selection of candidate genes to define these developmental stages in *Pinus* was partly limited by the few studies dealing with the molecular aspects of needle maturation [49] and also by the almost complete absence of full-length *Pinus radiata* sequences within public databases.

To firstly describe the different developmental stages and then to analyse the gene-specific regulation of needle maturation in *P*. *radiata*, we selected four genes which expression is characteristic of mature (Pr*RBCA*, Pr*RBCS*) and immature tissues (Pr*CSDP2*, Pr*SHMT4*), which are differentially expressed between growing and mature needles [12]. The study of Pr*RBCA* and Pr*RBCS* expression as a marker of photosynthetic capacity allowed the differentiation of the different stages of needle maturation. Interestingly, the expression level of these genes in B5 needles, in a transition between proliferative and expansive stages, was significantly higher than in B1 (+6.3-fold) and heterotrophic calli, and 7-fold lower than B12. Andriankaja et al. [5] established the importance of chloroplast differentiation as a regulator of the simultaneous onset of cell expansion and photosynthesis in Arabidopsis. In contrast to photosynthetic genes, the maximal expression of Pr*CSDP2* and Pr*SHMT4* was found in calli as these genes are reporters of undifferentiated tissues in Arabidopsis being also related to stress protection [35,36,50]. These genes were highly expressed in calli and were drastically reduced in expression in differentiated tissues. These results underline the suitability of this set of genes for studying the epigenetic processes that lead to specific gene regulation during needle development.

### Needle maturation correlates with changes in global DNA methylation, AcH4, H3K4me3 and H3K9me3 levels

The evolution of the global DNA methylation level during maturation and ageing of specific tissues, organs, and species has been previously reported [22,51–53]. In *Pinus*, different DNA methylation levels are associated with ageing and phase change [54], organ maturation [55], and stress response [56]. In contrast to these studies, which focused almost exclusively on DNA methylation and in the comparison of extreme situations, we analysed the progression of DNA methylation and four histone marks along a developmental gradient allowing the specific temporal dynamics to be described.

The degree of DNA methylation increased together with organ maturation, with the lowest level found in calli (de-differentiated tissue) and the highest in B12 needles. Surprisingly B1 and B5 needles showed the same methylation level, probably reflecting that in B5 needles late proliferative and early expansive stages are occurring at the same time requiring a large number of genes to be expressed. The plasticity of DNA methylation allows the quick change in the regulation of specific genes while maintaining the same global level, by interacting with different epigenetic mechanisms [57,58] and does not regulate all genes [25,52,59], with different transcriptional intensities at specific loci having the same global DNA methylation level.

Based on current knowledge [17,27,58,60,61] we selected four histone PTMs markers of gene activation (AcH4, H3K4me3) and repression (H3K9me3, H3K27me3). Less-differentiated tissue (B1, B5) showed a higher ratio of permissive/repressive marks compared to that of mature needles (B12) [55]. Similar to DNA methylation, B5 needles showed an unexpected abundance of AcH4 and H3K4me3 marks, maybe for allowing the transition between proliferative to expansive stages, in a similar way to bud reprogramming during flowering [62]. In contrast, fully mature B12 needles showed the highest abundance of the repressive mark H3K9me3. Finally, calli presented the lowest abundance of all histone marks, a striking phenomenon also described in Arabidopsis [63] and might counterbalance the low DNA methylation level. These data highlight the complexity of epigenetic networks that are extremely dynamic, with the relevance of each mechanism depending on the development stage and environmental condition.

Notably, the abundance of H3K27me3, considered one of the stronger marks for gene repression [26,60] and recently reported to be a critical mechanism for callus de-differentiation in Arabidopsis [63], was not significantly altered during needle ontology. Since H3K27me3 was shown to be a key regulatory mark for the regulation of photosynthesis genes we hypothesize that this mark is regulating different loci during development. Although global epigenetic levels are considered to be good biomarkers for key developmental processes [53,54] deeper epigenetic studies focusing on specific loci are necessary to fully understand development, stress response or environmental adaptions on forest species as a basis for improving forest productivity.

### The epigenetic marks H3K4me3 and H3K9me3 can be associated to the transcriptional activity of Pr*SHMT4* and, together with specific DNA methylation patterns, that of Pr*CSDP2*

The Pr*CSDP2* and Pr*SHMT4* genes constitute a good model for understanding the epigenetic changes associated with gene-silencing during needle development, since their expression is characteristic of calli and was progressively down-regulated during differentiation. The site-specific analysis of Pr*CSDP2* observed that different developmental stages showed different DNA methylation and histone signatures. This fact suggests that the chromatin topology of this locus changed during development, probably contributing to the different expression levels of Pr*CSDP2*. Changes in chromatin topology associated with gene expression levels have been extensively studied in animal systems [27,64].

The high reproducibility of bisulfite sequencing of first exon region of Pr*CSDP2* in samples from calli or B1 needles compared to other tissues could be related to the fact that these are less differentiated, with a small number of cell lines forming the different tissues. In spite of this, a clear increase in the proportion of methylated cytosines was observed from calli to mature needles. The promoter of this gene did not show any significant changes of cytosine methylation or histone modifications, possibly because it is a clear region surrounded by epigenetically regulated sequences. The epigenetic behaviour of this gene was similar to that reported for the *CSDP2* gene of Poplar (S4 Fig), in which the promoter had strong epigenetic signals more than 1 kb upstream and its first exon was more methylated in leaves than in buds [52]. Unfortunately, these high-throughput analyses do not achieve a single base resolution, which precludes further comparative analysis of the methylation of specific cytosines.

The different patterns of histone PTMs at the first exon of Pr*CSDP2* also highlighted the importance of the epigenetic regulation at this locus. In Arabidopsis, Charron et al. [32] provided a dataset in which H3K9me3 and H3K27me3 are associated with At*CSDP2* (AT4G38680) in expanded leaves, however, the dynamics of histone PTMs surrounding At*CSDP2* during leaf development have never been specifically studied. In this work, we found that the balance between AcH4, H3K4me3 and H3K9me3 is correlated to the expression level of this gene. Despite that, in callus, which showed the highest expression, there was a strong enrichment of H3K4me3 and AcH4, while B12 needles were characterized by a strong H3K9me3 signal. It seems that, in differentiated tissues, AcH4 is not playing a key role in the regulation of this gene, since the application of acetylant drugs (SAHA) did not increase gene expression. On contrary, in calli, both drugs induced gene expression maybe because the direct activation of this gene by SAHA and the downregulation of a hypothetical repressor by AnAc. This results points that there should be another mark, not present in needles, which allows effective gene expression in calli.

The enrichment of the H3K4me3 further indicates that this gene could be expressed widely [65], being in concordance to its cold protective function and its role during flowering and fruit development [36]. H3K27me3 was also associated with this locus in mature needles. This mark could not be detected in Arabidopsis using high throughput approaches, showing the importance of studying the complete epigenetic context to provide a reliable epigenetic analysis.

The promoter of Pr*SHMT4* does not possess cytosine-rich regions and it is not methylated in Poplar (POPTR_0017s08600) or Arabidopsis (AT4G13930), suggesting that its epigenetic regulation is based only in histone modifications. The expression of this gene was correlated to high levels of H3K4me3 and the presence of AcH4 in calli. Needles were characterized by a very low expression of this gene and the presence of H3K9me3 in its promoter, supporting the view of this modification as a strong repressive mark that is related to differentiation and maturity [66]. The unexpected presence of the repressive mark H3K27me3 in calli, underlines the complexity of the epigenetic network and suggests the need for site-specific studies to complement the available high-throughput models to provide an accurate analysis of chromatin microenvironments.

### Photosynthetic carbon fixation regulation is associated to a crosstalk between histone H4 acetylation and H3K9me3 at the promoter level

ChIP analyses revealed that the epigenetic regulation of Pr*RBCA* and Pr*RBCS* occurs via their promoters and not via their first exons, and could be mediated by interplay between AcH4 and H3K27me3. Despite the absence of specific studies of epigenetic regulation of photosynthesis and its chemical unmasking, these results are in agreement with the epigenetic changes observed during de-etiolation of Arabidopsis leaves [32] in which histone acetylation played an essential role for the activation of photosynthetic machinery. The reversibility of epigenetics could be closely related to the capacity for rapid adaptation or a return to the previous functional status of photosynthetic activities.

Furthermore, despite the maintenance of global H3K27me3 during development, this was a crucial mark for the repression of Pr*RBCA* and Pr*RBCS* in callus and during maturation in the case of *RBCS*. Genome-wide analysis of H3K27me3 in fully expanded leaves of Arabidopsis [26] supports our observations, since this modification was not found in At*RBCA* (AT2G39730) and in At*RBCS* (AT1G67090, AT5G38430, AT5G38420, AT5G38410). The developmental and tissue-specific transcriptional regulation of these genes justifies the absence of differential H3K4me3 patterns for genes with a low tissue specificity, proposed by [65].

As the promoters of Pr*RBCA* and Pr*RBCS* showed a low density of cytosine residues and consequently we suggested that DNA methylation does not have a direct regulatory function for these genes. This hypothesis is reinforced by the analysis of the Poplar [52] and Arabidopsis [59] leaf methylomes. In conclusion, we propose that histone acetylation and H3K27me3 specifically regulate the loci *RBCA* and *RBCS* in *P*. *radiata* and the promoter region is more responsive to epigenetic regulation than the first exon.

The similarities in the specific epigenetic regulation of *RBCA* and *RBCS* in different plant species suggest that this system has been conserved throughout evolution. However, further research is needed to increase our knowledge concerning the molecular biology of these mechanisms and their functional, ecological, and evolutionary significance.

## Supporting Information

S1 Fig Analysis of cytosine rich regions of the indicated genes. Observed vs Expected plot shows the ratio based on the frequency of C's and G's in that window. Percentage plot represents the rate of Cs and Gs within the studied sequence; putative islands plot indicates the region where cytosine rich region is predicted.
(PDF)

S2 Fig Representative blots showing the identification and quantification of (a) Acetylated Histone H4, (b) H3K27me3, (c) H3K9me3, and (d) H3K4me3 by immunobloting on protein extracts from calli (C), mature (B12), growing (B5), and immature (B1) needles. Band intensities were normalized against Tubulin (Tub).
(PDF)

S3 Fig Analysis of the Relative Quantity (RQ) of Pr*CSDP2*, Pr*RBCA*, Pr*RBCS*, and Pr*SHMT4* expression levels in (a) *P*. *radiata* calli and (b) needles after co cultivation with AnAc and SAHA. Expression levels were normalized to values in controls (untreated calli or needles). Different letters indicate significant differences between developmental stages (Tukey HSD test; p < 0.05).
(PDF)

S4 Fig Differential DNA methylation found in the promoter of the locus POPTR_0009s13460, ortholog of Pr*CSDP2*, when comparing leaf and apical buds. Poplar methylome [52] was visualized using gbrowse available at the Oregon State University (http://poplar.cgrb.oregonstate.edu/cgi-bin/gbrowse/Populus/; landmark: scaffold_9:10697662..10702661). The specific locus corresponding to POPTR_0009s13460 was defined after BLASTing the Pr*CSDP2* partial sequence to *Populus trichocarpa* genome v3 available at Phytozome (http://www.phytozome.net).
(PDF)

S1 File Fasta file containing the sequences of Pr*CSDP2*, Pr*SHMT4*, Pr*RBCA* and Pr*RBCS* promoters obtained by RACE and chromosome walking towards 5’ end. (FASTA)

S1 Table List of employed primers for qRT-PCR. (PDF)
