# Supplementary material for: Conserved Epigenetic Mechanisms Could Play a Key Role in Regulation of Photosynthesis and Development-Related Genes during Needle Development of Pinus radiata
Source: PLoS One. 2015 May 12;10(5):e0126405. doi: 10.1371/journal.pone.0126405 (PMC4429063; doi:10.1371/journal.pone.0126405)
Supplement: S1 Fig — Observed vs Expected plot shows the ratio based on the frequency of C's and G's in that window. Percentage plot represents the rate of Cs and Gs within the studied sequence; putative islands plot indicates the region where cytosine rich region is predicted. (PDF) [file pone.0126405.s001.pdf]

Figure S1:

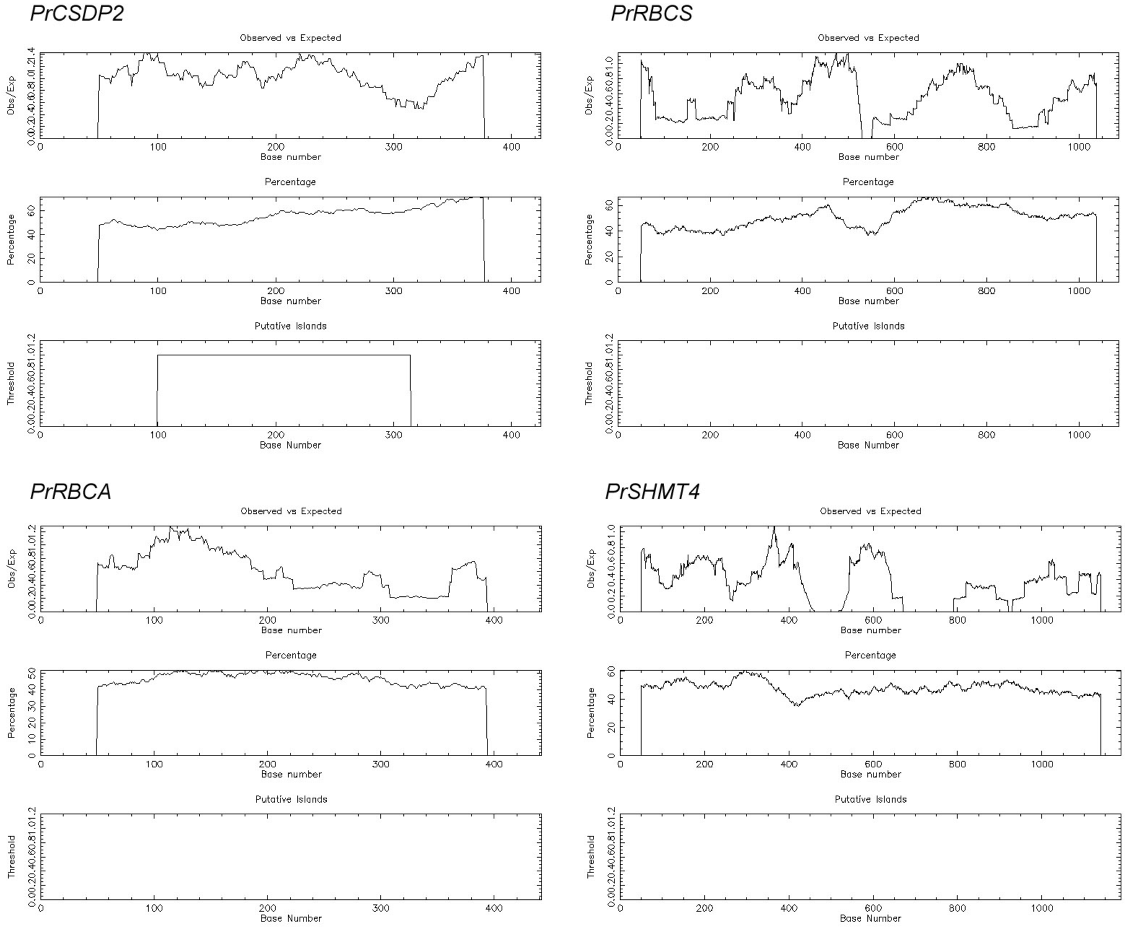

Figure S1: Analysis of cytosine rich regions of the indicated genes. Observed vs Expected plot shows the ratio based on the frequency of C's and G's in that window. Percentage plot represents the rate of Cs and Gs within the studied sequence; putative islands plot indicates the region where cytosine rich region is predicted.
