# Supplementary material for: Conserved Epigenetic Mechanisms Could Play a Key Role in Regulation of Photosynthesis and Development-Related Genes during Needle Development of Pinus radiata
Source: PLoS One. 2015 May 12;10(5):e0126405. doi: 10.1371/journal.pone.0126405 (PMC4429063; doi:10.1371/journal.pone.0126405)
Supplement: S2 Fig — Band intensities were normalized against Tubulin (Tub). (PDF) [file pone.0126405.s002.pdf]

Figure S2

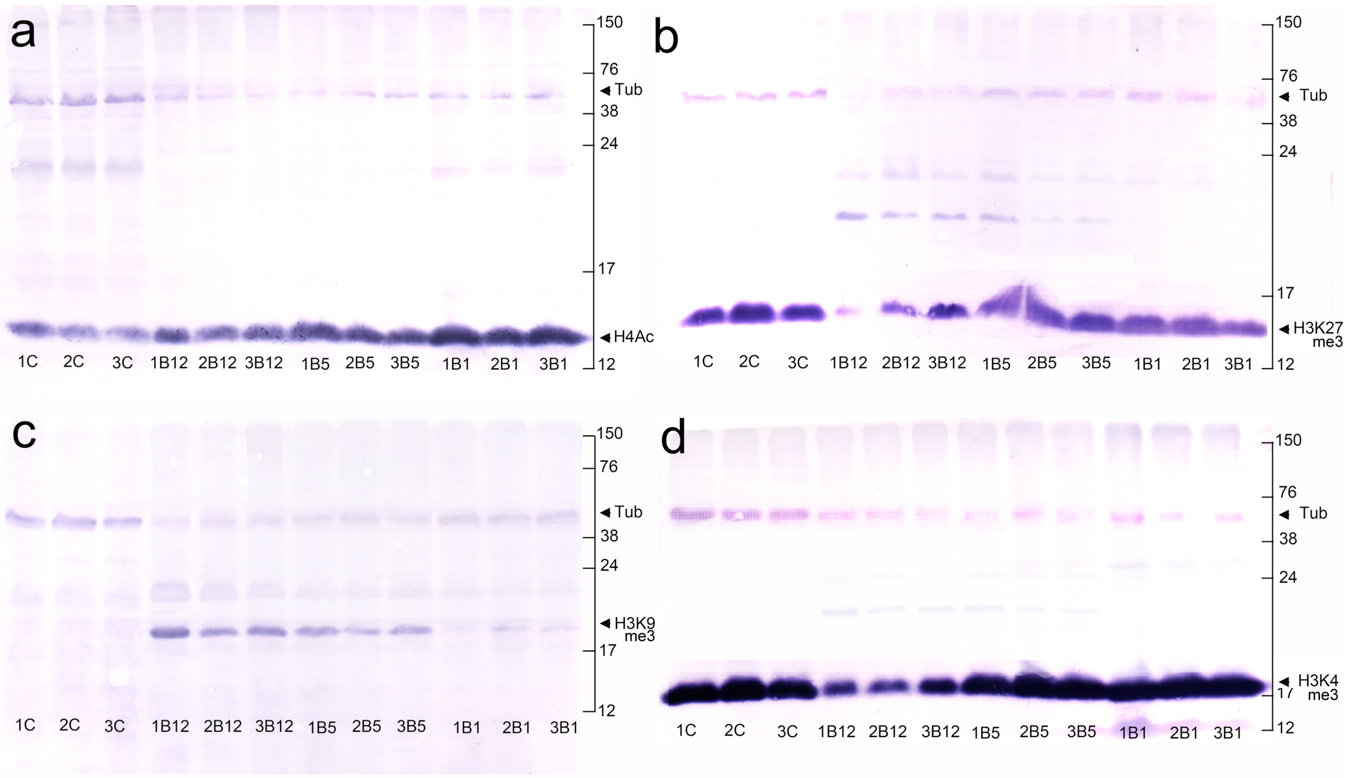

Figure S2: Representative blots showing the identification and quantification of (a) Acetylated Histone H4, (b) H3K27me3, (c) H3K9me3, and (d) H3K4me3 by immunobloting on protein extracts from calli (C), mature (B12), growing (B5), and immature (B1) needles. Band intensities were normalized against Tubulin (Tub).
