# Supplementary material for: Conserved Epigenetic Mechanisms Could Play a Key Role in Regulation of Photosynthesis and Development-Related Genes during Needle Development of Pinus radiata
Source: PLoS One. 2015 May 12;10(5):e0126405. doi: 10.1371/journal.pone.0126405 (PMC4429063; doi:10.1371/journal.pone.0126405)
Supplement: S3 Fig — Different letters indicate significant differences between developmental stages (Tukey HSD test; p < 0.05). (PDF) [file pone.0126405.s003.pdf]

Figure S3

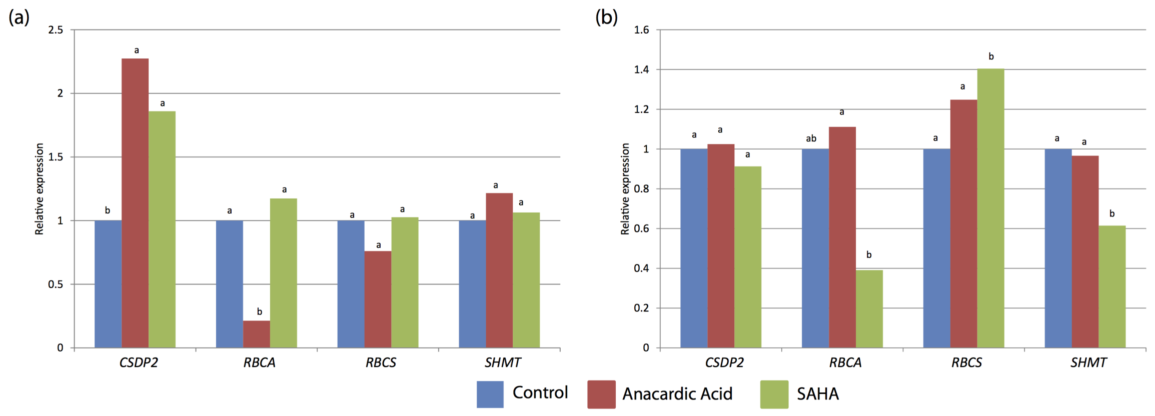

Figure S3: Analysis of the Relative Quantity (RQ) of Pr*CSDP2,* Pr*RBCA*, Pr*RBCS*, and Pr*SHMT4* expression levels in (a) P. *radiata calli* and (b) needles after co cultivation with AnAc and SAHA. Expression levels were normalized to values in controls (untreated calli or needles). Different letters indicate significant differences between developmental stages (Tukey HSD test; $p < 0.05$).
