# Supplementary material for: Conserved Epigenetic Mechanisms Could Play a Key Role in Regulation of Photosynthesis and Development-Related Genes during Needle Development of Pinus radiata
Source: PLoS One. 2015 May 12;10(5):e0126405. doi: 10.1371/journal.pone.0126405 (PMC4429063; doi:10.1371/journal.pone.0126405)
Supplement: S4 Fig — Poplar methylome [52] was visualized using gbrowse available at the Oregon State University (http://poplar.cgrb.oregonstate.edu/cgi-bin/gbrowse/Populus/; landmark: scaffold_9:10697662..10702661). The specific locus corresponding to POPTR_0009s13460 was defined after BLASTing the PrCSDP2 partial sequence to Populus trichocarpa genome v3 available at Phytozome (http://www.phytozome.net). (PDF) [file pone.0126405.s004.pdf]

Figure S4

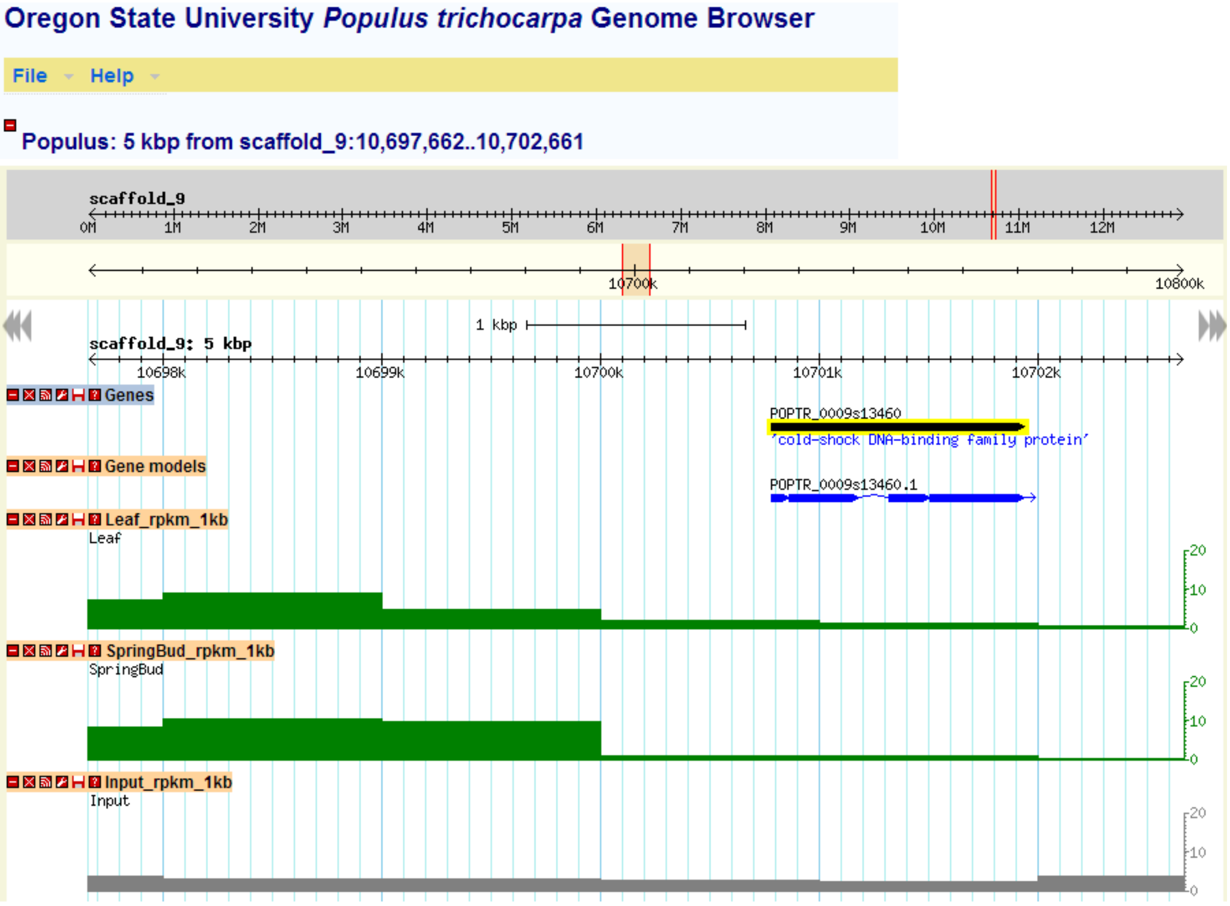

Figure S3: Differential DNA methylation found in the promoter of the locus POPTR_0009s13460, ortholog of Pr*CSDP2*, when comparing leaf and apical buds. Poplar methylome (Vining *et al.*, 2012) was visualized using gbrowse available at the Oregon State University (http://poplar.cgrb.oregonstate.edu/cgi-bin/gbrowse/Populus/; landmark: scaffold_9:10697662..10702661). The specific locus corresponding to POPTR_0009s13460 was defined after BLASTing the Pr*CSDP2* partial sequence to *Populus trichocarpa* genome v3 available at Phytozome (http://www.phytozome.net).

References:

Vining K, Pomraning K, Wilhelm L, Priest H, Pellegrini M, Mockler T, Freitag M, Strauss S. 2012. Dynamic DNA cytosine methylation in the Populus trichocarpa genome: tissue-level variation and relationship to gene expression. *BMC Genomics* **13**(1): 27.
