# Supplementary material for: Conserved Epigenetic Mechanisms Could Play a Key Role in Regulation of Photosynthesis and Development-Related Genes during Needle Development of Pinus radiata
Source: PLoS One. 2015 May 12;10(5):e0126405. doi: 10.1371/journal.pone.0126405 (PMC4429063; doi:10.1371/journal.pone.0126405)
Supplement: S1 Table — (PDF) [file pone.0126405.s006.pdf]

**Table S1:** List of employed primers for Bisulfite sequencing, qRT-PCR, and ChIP analyses.

| **Gene** | **Forward (5'->3')** | **Reverse (5'->3')** | **Tm** |
|---|---|---|---|
| | **Bisulfite sequencing** | | |
| Pr*CSDP2*-Outer | GGGTTATGGTTTTATAATTTYTGATG | TCCCACCCACCRCCACC | 60 |
| Pr*CSDP2*-Inner | CCRRAAAGGTTAAGTGGTT | RAACCACCACCTCTTCRATTC | 52 |
| | **RT-qPCR** | | |
| *18S* | GCGAAAGCATTTGCCAAGG | ATTCCTGGTCGGCATCGTTTA | 58 |
| *ACTIN* | CACTGCACTTGCTCCCAGTA | AACCTCCGATCCAAACACTG | 58 |
| *GAPDH* | AAGATCCTCGGGAAAGGAGA | CTTCCTTGCTCAATGCAACA | 58 |
| Pr*RBCS* | GGAACCATCTCGAGGAGGTA | CTCTGTGCACCCAACATC | 58 |
| Pr*RBCA* | CAAGGAGCAATAGGCATCTA | AGATGAGCCCAGTCTTTCTC | 58 |
| Pr*SHM4* | AACTGCGTTCTTGTTGATTG | CTTGATGAACGAGGGATACA | 58 |
| Pr*CSDP2* | TTTCATAACCCCTGATGATG | ATCTAGGGCCTTGGTTCTTC | 58 |
| | **ChIP** | | |
| Pr*CSDP2 Promoter* | ATTAATAATAAAGCACATGGGGAGAAATAA | AAATATATGTACGTATGGTAGAAAGGAGGT | 60 |
| Pr*CSDP2 1st Exon* | GAGGGATTCGAACCATGTCTG | CGTGTACTCGACGGCCTCT | 60 |
| Pr*RBCA Promoter* | ACTGTTAGCTCCATCACTGTCTCC | GCTTGCTCTCATCAATTTCAGC | 60 |
| Pr*RBCA 1st Exon* | TTTTTCCTATATAATCAGAAGCAATAACTG | TAAATACATAATTGAGGAGCTCAGTAAGTC | 60 |
| Pr*RBCS Promoter* | GTGAGCTGCACCAGACAAAGC | TCTCAGGTGAGTAACCAGCATCACG | 61 |
| Pr*RBCS 1st Exon* | AAGACCAGCAATGGCAAGGGGA | TTCCGCTGCTGCGTTGCTCA | 60 |
| Pr*SHMT Promoter* | AGAAGCCGGTTTCCTAGCGCAA | GAAGAGAGGCTGGGATCGTGT | 60 |
| Pr*SHMT 1st Exon* | TTTTCATCTGGGCATCACTAGTTT | TTGCCTTCTCTTCTCCTTCTCAAT | 62 |
